# The proatherosclerotic function of indoleamine 2, 3-dioxygenase 1 in the developmental stage of atherosclerosis

**DOI:** 10.1038/s41392-019-0058-5

**Published:** 2019-07-19

**Authors:** Heng Liang, Mantian Chen, Fangfei Qi, Lei Shi, Zhenzhen Duan, Ruoyu Yang, Jinchao He, Bin Lou, Yigang Li, Qing Yang

**Affiliations:** 1 State Key Laboratory of Genetic Engineering, Department of Biochemistry, School of Life Sciences, Fudan University, Shanghai, China; 2 Department of Cardiovascular Diseases, Xinhua Hospital, School of Medicine, Shanghai Jiaotong University, Shanghai, China; 3 School of Pharmacy, Fudan University, Shanghai, China

**Keywords:** Cardiology, Molecular biology

## Abstract

The discrepancy of indoleamine 2, 3-dioxygenase 1 (IDO1) function in atherosclerosis has been noted. Compared to the protective effect of IDO1 against established atherogenesis, the role of IDO1 in the developmental process of atherosclerosis is still unclear. Here, the expression patterns and activities of IDO1 and its isoenzyme tryptophan 2,3-dioxygenase (TDO) in aortas and blood samples of patients with atherosclerosis were investigated. IDO1 and TDO were colocalized with CD3-positive lymphocytes and CD68-positive macrophages in atherosclerotic lesions. The expression and activity of IDO1 and TDO increased with the grade of the histological classification in early atherosclerosis (grade I, II), but the increase did not continue in advanced atherosclerosis (grade III). Treatment of THP-1 macrophages (THP-M) with oxidized low-density lipoprotein (oxLDL) induced the expression of IDO1 via the PI3K/Akt/NF-κB pathway, indicating the potential function of IDO1 in foam cells. Before and after treatment with oxLDL on THP-M, IFN-γ-induced IDO1 exhibited different degrees of promotion on foaming, inflammatory factor production and cell apoptosis. Finally, we found that the IDO1 inhibitor 1-methyl-tryptophan could elevate the high-density lipoprotein cholesterol level in serum and reduce the area of the aortic atherosclerotic lesions in high-fat diet-fed ApoE^−/−^ mice. Our study indicated that IDO1 played a complicated and unfixed role in the entire process of atherogenesis, despite the atheroprotective role in established atherosclerosis. IDO1 also had proatherosclerotic functions in the developmental stages of atherosclerosis. Modulation of IDO1 could be a good method for alleviating atherosclerosis.

## Introduction

Atherosclerosis is an arterial disease involving progressive accumulation of cholesterol deposits and eventual buildup of plaque inside the arterial wall.^1^ Atherosclerosis is associated with inflammation, abnormal lipid metabolism, damage to the vascular endothelial structure and immune regulation.^2^ The exact cause of atherosclerosis is not known, although hypertension, diabetes, smoking, oxidized low-density lipoprotein (oxLDL) and so forth are known to be risk factors of atherosclerosis.^3–5^ Foam cell formation due to excessive accumulation of cholesterol by macrophages is a pathological hallmark of atherosclerosis.^6,7^ During atherogenesis progression, circulating monocytes adhere to the intima and differentiate into macrophages that phagocytize oxLDL via scavenger receptors, thereby transforming into foam cells.^8^ oxLDL can induce apoptosis,^9^ inflammatory responses and phagocytosis of macrophages.^10–12^

The activated kynurenine pathway (KP) has been suggested to play an important role in atherogenesis. KP is the primary pathway of tryptophan (Trp) catabolism in most mammalian cells^13,14^ and generates several bioactive catabolites, such as kynurenine (Kyn), anthranilic acid (AA), kynurenic acid (KA), 3-hydroxykynurenine (3-HK), xanthurenic acid (XA), and 3-hydroxyanthranilic acid (3-HAA).^15–17^ Abnormal levels of KA and 3HAA have been demonstrated in atherosclerosis. High concentrations of KA in atheromatous plaques were associated with an unstable plaque phenotype in human atherosclerotic lesions, whereas there were no detectable or low levels of KA in stable fibrous plaques.^18^ Elevation of 3-HAA levels by IDO1 activation inhibited vascular inflammation and subsequent atherosclerosis in LDLR^−/−^ or ApoE^−/−^ mice. Furthermore, 3-HAA can block atherogenesis.^19,20^ In addition, a synthetic derivative of AA, 3, 4-dimethoxycinnamoyl anthranilic acid (3, 4-DAA), has been reported to inhibit inflammation and lesion formation after perivascular collar-mediated arterial injury in ApoE^−/−^ mice.^21^

Indoleamine 2, 3-dioxygenase (IDO1), the first and rate-limiting enzyme of the KP, has also been investigated due to its role in the pathogenic process of atherosclerosis.^22^ IDO1 is expressed in a variety of cells, including endothelial cells,^23–25^ vascular smooth muscle cells, macrophages,^26^ leukocytes,^27^ and dendritic cells (DCs),^28^ all of which are present in the artery wall. IDO1 can be induced by proinflammatory modulators, such as IFN-γ^29^ and Toll-like receptor ligands.^30^ IDO1 expression in endothelial cells and myeloid can mediate endothelium-dependent vasodilation and immune regulation, respectively.^31,32^ The functions of IDO1 in atherogenesis are complicated and inconsistent. In the Tampere Vascular Study, increased IDO1 expression was observed in the macrophage-rich cores of human atherosclerotic plaques.^26^ In a large cohort study, IDO1 activity was found to have a significant positive correlation in both sexes with carotid artery intima/media thickness, an early marker of atherosclerosis.^33^ Specifically, IDO1 activity has been reported to positively correlate with a range of atherosclerosis risk factors for the female population, including age, BMI, and LDL cholesterol.^34^ In contrast, the atheroprotective role of IDO1 has also been documented. IDO1-deficient ApoE^−/−^ mice (ApoE^−/−^Indo^−/−^) developed larger atherosclerotic lesions and an unfavorable lesion phenotype.^21,35^ In addition, inhibition of IDO1 with the IDO1 inhibitor 1-methyl tryptophan (1-MT) led to increased vascular inflammation and aggravated atherosclerosis in ApoE^−/−^ mice.^36^ Tryptophan 2,3-dioxygenase (TDO) is an isozyme of IDO1. TDO mainly exists in the liver and brain and mediates basal KP.^37^ The activities of IDO1 and TDO are usually represented by the Kyn/Trp ratio.^38^ TDO can be induced by glucocorticoids, both endogenously through stress and exogenously by dexamethasone.^39^ Unlike IDO1, the involvement of TDO in atherogenesis has rarely been described.

In this study, we investigated the expression patterns and activities of IDO1 and TDO in aortas and blood samples of patients with atherosclerosis. Using THP-M, the effects of oxLDL on IDO1 expression and the related PI3K/Akt/NF-κB pathway^40–42^ were studied. Using THP-M, the effects of IDO1 on the degree of foaming, inflammatory factor production and apoptosis before and after oxLDL treatment were also explored. Using ApoE^−/−^ mice, the therapeutic effect of the IDO1 inhibitor 1-MT was explored.

## Results

### IDO1 and TDO were expressed in atherosclerotic lesions and codistributed with CD3-positive lymphocytes and CD68-positive macrophages

**Fig. 1:**
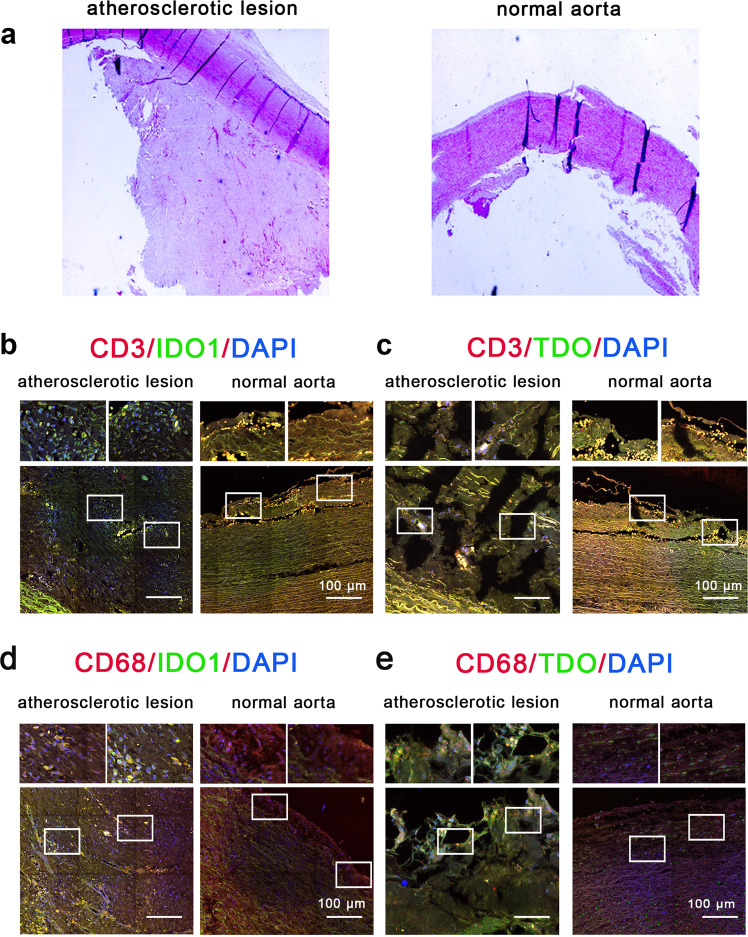
IDO1 and TDO were expressed in atherosclerotic lesions and codistributed with CD3-positive lymphocytes and CD68-positive macrophages in patients with advanced atherosclerosis. **a** Pathological changes of the coronary artery were revealed by HE staining (×400). **b**, **c** IDO1, TDO and the lymphocyte marker CD3 were detected in atherosclerotic lesions by immunostaining analysis (×400). **d**, **e** IDO1, TDO and the monocyte marker CD68 were detected in atherosclerotic lesions by immunostaining analysis (×400). The top panel in **b**–**e** exhibits an enlarged view of the representative region in the bottom panel

In the Tampere Vascular Study, IDO1 expression was observed in the macrophage cores of human atherosclerotic plaques.^26^ Here, to clarify the potential expression of IDO1 and TDO in advanced atherosclerotic lesions in human patients, immunohistochemistry was performed. As shown in Fig. 1a (right), in the normal aortic vascular wall, the structures of the tunica intima, tunica media and tunica adventitia were clear and in good order. Cells such as smooth muscle cells in the normal aortic vascular wall were arranged neatly, and no obvious inflammatory cell infiltration was observed. However, in the atherosclerotic aorta wall (Fig. 1a, left), lesions were formed at the tunica intima, and the vascular wall structure was not clear and thicker than the normal structure. In the atherosclerotic aortic plaque, the elastic plate was disorganized, broken or disappeared, the distribution of smooth muscle cells was abnormal and atrophic, and diffused inflammatory cell infiltration appeared. Thus, these paraffin-embedded human aortic samples were available for future immunostaining studies. The immunostaining results showed that there were almost no IDO1-positive or TDO-positive cells in the normal aorta. IDO1 and TDO were found to be expressed in atherosclerotic lesions and codistributed with CD3-positive lymphocytes and CD68-positive macrophages (Fig. 1b-e). In a previous report,^26^ immunohistochemistry analysis demonstrated that IDO1 was expressed in the atheromatous core and codistributed with monocyte-macrophages (CD68-positive cells). Herein, for the first time, we found that IDO1 and TDO were codistributed with lymphocytes (CD3-positive cells). CD3-Ab treatment was reported to induce rapid regression of established atherosclerosis via reducing CD4^+^ T cells and increasing the proportion of regulatory T cells (Tregs),^43^ which indicated the function of CD3-positive T cells in atherosclerosis. The finding of the localization of IDO1 and TDO with CD3-positive T cells suggested the involvement of IDO1 and TDO in this T cell-mediated immune response in atherosclerosis. In addition, the expression of TDO in paraffin-embedded human aortic samples has not been reported before.

### The expression and activity of IDO1 and TDO varied based on the grade of the histological classification of atherosclerosis patients

**Fig. 2:**
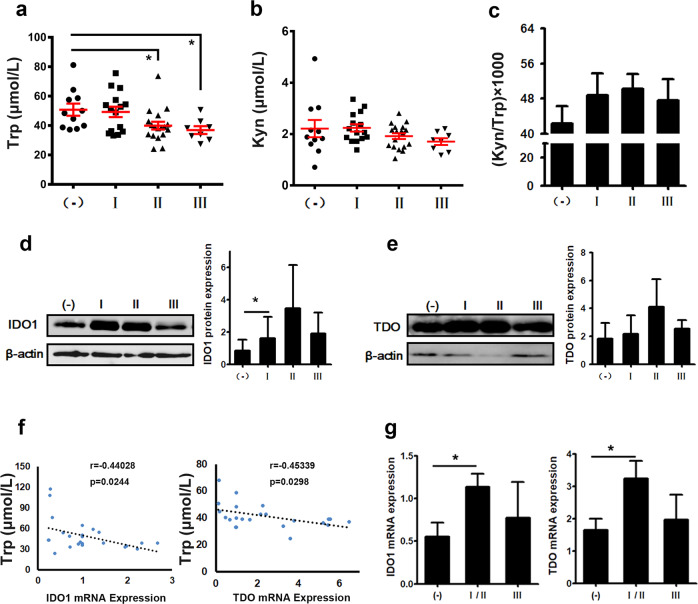
The expression and activity of IDO1 and TDO in atherosclerotic blood samples. Serum concentrations of tryptophan (**a**) and kynurenine (**b**) and the kynurenine to tryptophan ratio Kyn/Trp (**c**) in blood samples of atherosclerotic patients with different histological classification grades ((−): *n* = 10, I: *n* = 16, II: *n* = 18, III: *n* = 7). **d**, **e** IDO1 and TDO protein levels were determined by western blot assays. **f** Associations between the expression of IDO1 and TDO and the concentration of Trp (*r* = −0.44028, *p < 0.05; r = −0.45339, *p < 0.05). **g** IDO1 and TDO mRNA expression was determined by real-time PCR ((−): *n* = 6, I/II: *n* = 12, III: *n* = 4). Data were based on at least three independent experiments. *p < 0.05

We examined the concentrations of Trp (Fig. 2a) and Kyn (Fig. 2b) and calculated the ratio of Kyn/Trp (Fig. 2c) of the blood samples of atherosclerosis patients at different histological classification grades (grade I–II is early atherosclerosis, and grade III is advanced atherosclerosis and that post-myocardial infarction). Compared to the normal group, the concentrations of Trp slightly decreased in the grade I group and almost decreased by 50% in the grade II group. However, the concentration of Trp in the grade III group decreased markedly compared with that of the normal group, but it was not significantly lower than that of the grade II group (Fig. 2a). There was no significant difference between the concentrations of Kyn in the different groups, although the concentration of Kyn in the grade I group was slightly higher than that in the normal group (Fig. 2b). The ratio of Kyn/Trp in pathological groups (grade I–III) was higher than that of the normal group, and the value of the grade I/II group was higher than that of the grade III group (Fig. 2c). Thus, the activity of IDO1 and TDO increased in early atherosclerosis (grade I–II); however, the increase did not continue in advanced atherosclerosis (grade III).

The IDO1 protein expression in the pathological groups was higher than that in the normal group. The highest expression was achieved in grade II other than grade III (Fig. 2d). A similar result was observed in TDO protein expression (Fig. 2e). Furthermore, significant correlations between the Trp concentration and IDO1 mRNA expression (Trp vs. IDO1: *r* = −0.44028, *p* < 0.05; Fig. 2f) or TDO mRNA expression (Trp vs. TDO: *r* = −0.45339, *p* < 0.05; Fig. 2f) were clarified. IDO1 mRNA expression in the grade I/II group was 2 times higher than that in the normal group and was also higher than that in the grade III group. IDO1 mRNA expression in the grade III group was slightly higher than that of the normal group. TDO mRNA expression showed a similar variation profile (Fig. 2g).

From the result, we found that, generally, the expression and activity of IDO1 and TDO tended to increase with the grade of the histological classification in early atherosclerosis (grade I, II), but the increase did not continue in advanced atherosclerosis (grade III). Compared to that of the early atherosclerosis stage, the expression and activity of IDO1 and TDO were downregulated in the advanced atherosclerosis stage. Given the atheroprotective role of IDO1 in established atherosclerosis, the comparable downregulation of IDO1 and TDO in advanced atherosclerosis may lead to post myocardial infarction.

### oxLDL induced IDO1 expression via the PI3K/Akt/NF-κB pathway in THP-M

**Fig. 3:**
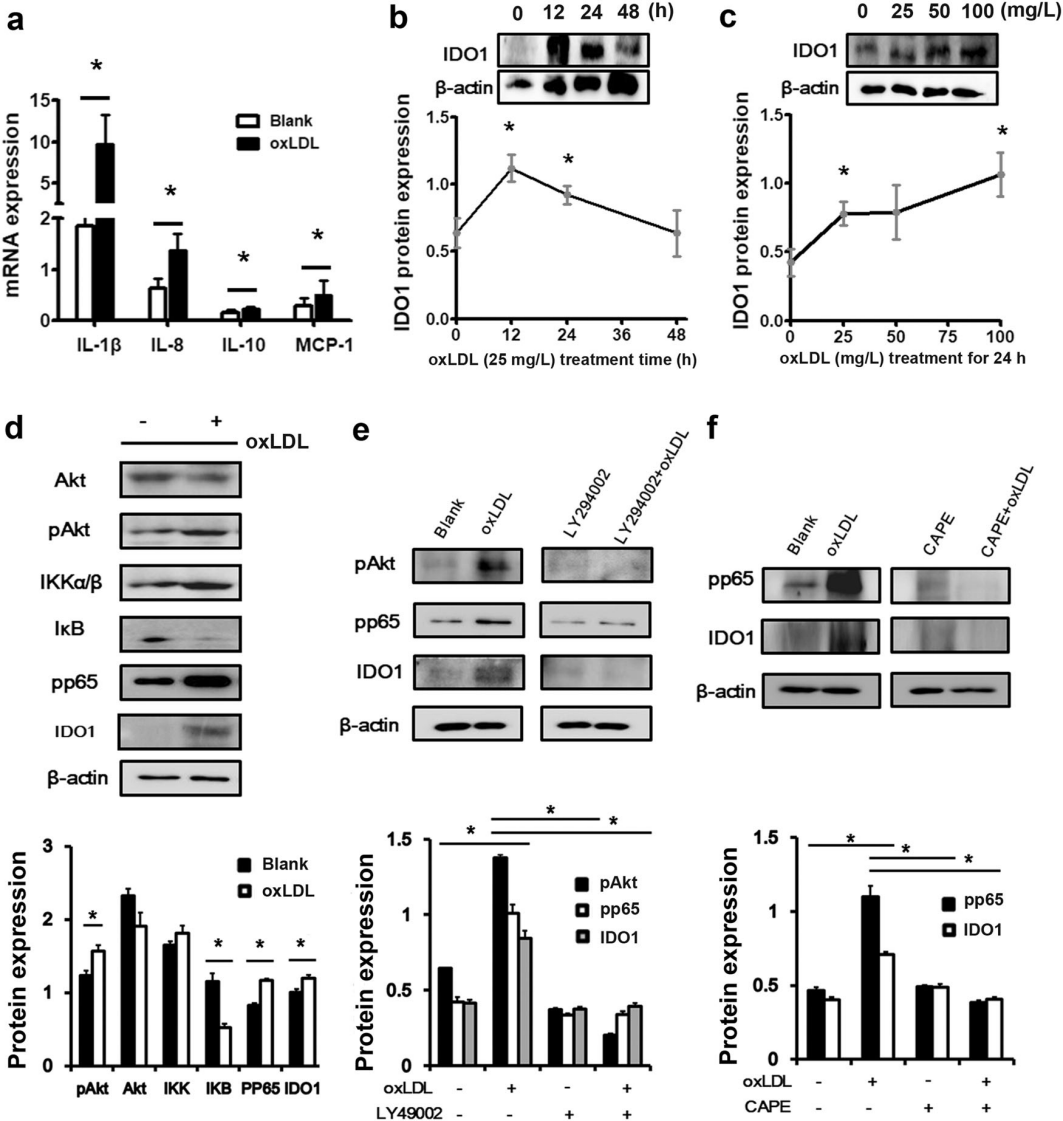
oxLDL induced IDO1 expression via the PI3K/Akt/NF-κB pathway in THP-M. **a** oxLDL induced the production of inflammatory factors. The mRNA levels of IL-10, MCP-1, IL-1β and IL-8 were measured by real-time PCR. **b** THP-M were challenged with 25 mg/L oxLDL for 12, 24, and 48 h, and IDO1 expression was detected by western blot analysis. **c** THP-M were treated with oxLDL for 24 h at 0, 25, 50 and 100 mg/L, and IDO1 expression was detected by western blot analysis. **d** The expression of PI3K/Akt/NF-κB pathway-related proteins in THP-M was detected by western blot analysis. THP-M were treated with oxLDL (25 mg/L) for 24 h. **e** The expression levels of pAkt, pp65, IDO1 and β-actin were detected by western blots. THP-M were pretreated with the Akt inhibitor LY294002 (5 μM) for 24 h and then treated with oxLDL (25 mg/L) for 24 h. Data were based on at least three independent experiments. *p < 0.05. **f** The expression levels of pp65, IDO1 and β-actin were detected by western blots. THP-M were pretreated with the NF-κB inhibitor CAPE (50 μM) for 24 h and then treated with oxLDL (25 mg/L) for 24 h

Given that the expression and activity of IDO1 varied with the different grades of the histological classification of atherosclerosis, we sought to clarify the involvement of IDO1 in established atherogenesis. THP-M foam cell formation induced by oxLDL is a marker of established atherogenesis because macrophage foaming is a pathological hallmark of atherosclerosis.^44^ Therefore, we investigated the effect of oxLDL on IDO1. First, the influence of oxLDL on the production of some inflammatory factors, including IL-1β, IL-8, MCP-1, and IL-10, was studied because inflammatory factors are important markers of foam cells and can be used to evaluate the degree of foaming.^26^ The levels of these inflammatory factors, especially IL-1β and IL-10, were found to be elevated upon treatment with oxLDL (Fig. 3a). Next, the effects of oxLDL treatment at different times and doses on IDO1 expression were evaluated. The highest IDO1 expression was achieved by 12 h of treatment with 25 mg/L oxLDL and then decreased with time (Fig. 3b). Different concentrations of 0, 25, 50, and 100 mg/L oxLDL treatment for 24 h led to a dose-dependent increase in IDO1 expression (Fig. 3c). IDO1 expression is regulated by the NF-κB pathway, which can be activated by the PI3K/Akt signaling pathway.^41,45^ In addition, inhibition of the PI3K/Akt signaling pathway can downregulate the expression of NF-κB to ameliorate the progression of atherosclerosis.^42,46–48^ However, in atherosclerosis, whether the PI3K/Akt/NF-κB signaling pathway modulates IDO1 is unclear.

As shown in Fig. 3d, under the treatment of oxLDL, the expression levels of IDO1, pAkt, IKKα/β, and pp65 were significantly enhanced, implying that the PI3K/Akt/NF-κB pathway was activated to upregulate the expression of IDO1. pAkt and IKKα/β/pp65 are classical markers for the PI3K/Akt and NF-κB pathways, respectively. When the specific NF-κB inhibitor CAPE was used, the NF-κB signaling pathway was inhibited and IDO1 expression was decreased. Treatment with oxLDL did not remarkably upregulate IDO1 expression when the NF-κB pathway was inhibited (Fig. 3f). A similar result was observed when the specific PI3K/Akt inhibitor LY294002 was used (Fig. 3e). In conclusion, for the first time, oxLDL was shown to induce IDO1 expression via the PI3K/Akt/NF-κB pathway in THP-M. This result also indicated the involvement of IDO1 in foam cells and established atherogenesis.

### Before and after oxLDL treatment, IFN-γ-induced IDO1 exhibited different degrees of promotion on foaming

**Fig. 4:**
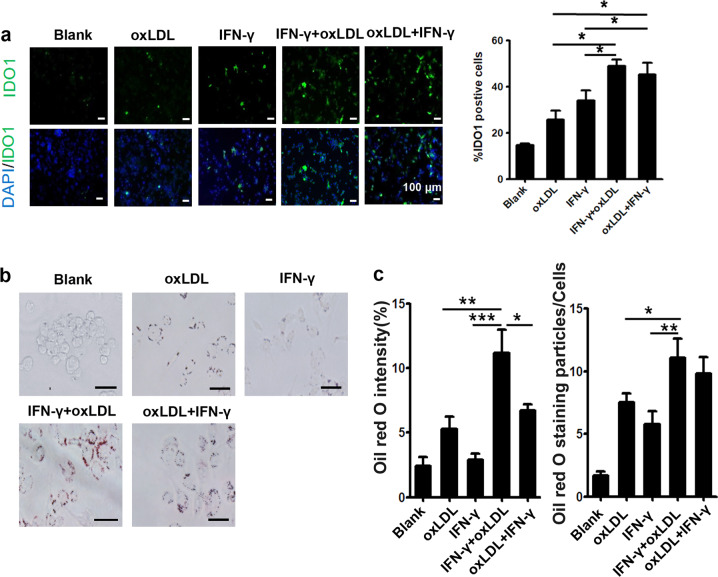
Before and after oxLDL treatment, the promoting effects of IFN-γ on IDO1 expression and the degree of foaming of THP-M. **a** Induction of IDO1 by different treatments was detected by immunofluorescence (×200). **b** The degree of foaming was detected by Oil red O staining (×400). **c** Quantitative analysis of Oil red O intensity (Oil red O staining area/cell area and Oil red O staining particles per cell). The designation of different treatments, such as oxLDL, IFN-γ, IFN-γ+ oxLDL and oxLDL+IFN-γ, was described in the Materials and Methods. Data were based on at least three independent experiments. *p < 0.05, **p < 0.01, ***p < 0.001

Given the involvement of IDO1 in established atherosclerosis, we attempted to clarify the role of IDO1 in the developmental process of atherosclerosis. Herein, the transformation of THP-M to foam cells was used to imitate the developmental process of atherosclerosis. In the present study, we designated several treatments as “oxLDL”, “IFN-γ”, “oxLDL+IFN-γ”, and “IFN-γ+oxLDL” and investigated their effects on IDO1 and the degree of foaming. The immunofluorescence study (Fig. 4a) showed that oxLDL slightly increased IDO1 expression, while IFN-γ significantly induced IDO1 expression. Two different treatments, “oxLDL+IFN-γ” and “IFN-γ+oxLDL”, induced IDO1 expression to a similar extent. The combined treatment of oxLDL and IFN-γ induced IDO1 expression to a greater degree than the single treatment of oxLDL or IFN-γ. Western blot analysis showed the same results (Supplementary Fig. S1). A similar result was observed in IDO1 activity (Kyn/Trp) (Table 1). Compared with the “blank” group, the “oxLDL” group showed significant Oil red O staining, indicating the formation of foam cells. However, the “IFN-γ” group showed similar Oil red O staining to the “blank” group, which indicated that IFN-γ could not transform THP-M into foam cells without oxLDL. This finding was in line with experimental data on the synergism of inflammation and a high-fat diet in inducing metabolic changes in mice.^49^ Compared with the “oxLDL” group, the “IFN-γ+oxLDL” group showed stronger Oil red O staining, which indicated that IFN-γ could promote the transformation to foam cells (Fig. 4b, c). Notably, the Oil red O staining of the “IFN-γ+oxLDL” group was deeper than that of the “oxLDL +IFN-γ” group (Fig. 4b, c).

**Table 1:** Concentrations (mean ± SD) of Trp and Kyn and the Kyn/Trp ratio in the supernatant of THP-M

	Blank	oxLDL	IFN-γ	IFN-γ+oxLDL	oxLDL+IFN-γ
Trp (μmol/L)	41.58 ± 10.56	31.27 ± 13.28	23.92 ± 9.92	16.62 ± 10.91	23.04 ± 5.66
Kyn (μmol/L)	2.40 ± 1.14	2.91 ± 0.15	4.31 ± 2.50	10.93 ± 7.77	10.67 ± 1.84
(Kyn/Trp) × 100	5.54 ± 1.60	11.28 ± 6.96	17.12 ± 5.32	66.12 ± 34.76*	50.11 ± 23.17

Data were based on at least three independent experiments

*p < 0.05

Thus, IFN-γ significantly induced the expression and activity of IDO1 in THP-M. Before oxLDL treatment (before foam cell formation, before atherosclerosis was established), IFN-γ-induced IDO1 exhibited greater promotion on the degree of foaming than that after oxLDL treatment (after foam cell formation, after atherosclerosis was established).

**Fig. 5:**
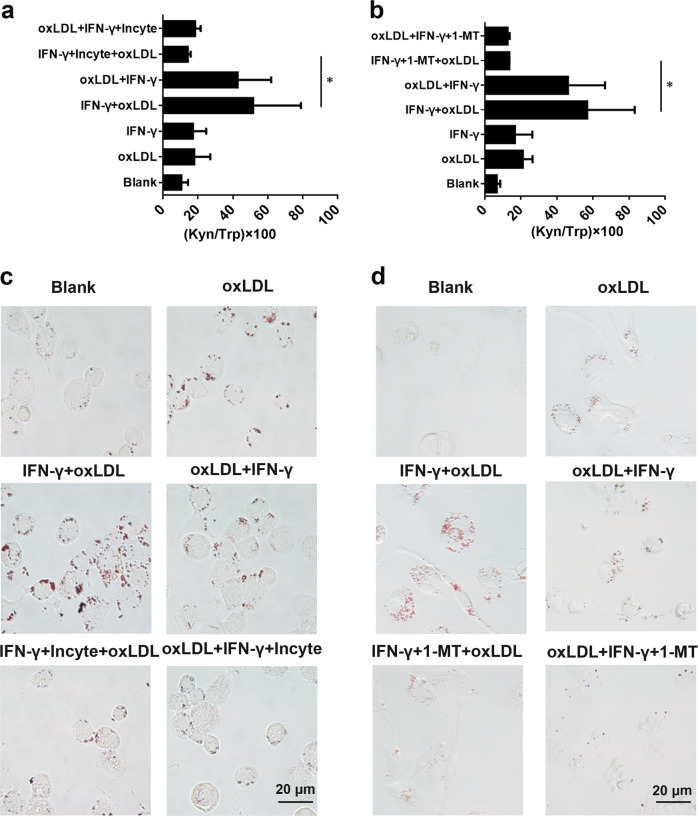
Before and after oxLDL treatment, the IFN-γ-mediated promotion of THP-M foaming was reversed by IDO1 inhibition. **a**, **b** IDO1 activity (Kyn/Trp) was detected by HPLC. **c**, **d** The results of Oil red O staining (×400). The designation of different treatments, such as oxLDL, IFN-γ, IFN-γ+oxLDL, oxLDL+IFN-γ, IFN-γ+Incyte+oxLDL, oxLDL+IFN-γ+Incyte, IFN-γ+1-MT+oxLDL and oxLDL+IFN-γ+1-MT, was described in the Materials and Methods. Data were based on at least three independent experiments. *p < 0.05

To further confirm the involvement of IDO1 in THP-M foaming, the effect of IDO1 inhibition in this matter was investigated. First, the inhibitory effects of the IDO1 inhibitor Incyte or 1-MT on IDO1 activity were tested (Fig. 5a, b). The single treatments of “IFN-γ” and “oxLDL” slightly, while the combination treatments of “IFN-γ+oxLDL” and “oxLDL+IFN-γ” markedly, increased the IDO1 activity (Kyn/Trp ratio), which was consistent with the result of Table 1. The enhanced IDO1 activity produced by “IFN-γ+ oxLDL” and “oxLDL+ IFN-γ” treatment was reversed by supplementation with Incyte or 1-MT, regardless of the order of treatment, before or after the oxLDL treatment (Fig. 5a, b). In addition, the formation of foam cells was affected by addition of IDO1 inhibitors. As shown in Fig. 5c, the Oil red O staining of the “IFN-γ+Incyte+oxLDL” group was lighter than that of the “IFN-γ+oxLDL” group and was similar to that of the “oxLDL” group. The Oil red O staining of the “oxLDL+IFN-γ+Incyte” group was lighter than that of the “oxLDL+ IFN-γ” group and was almost the same as that of the “oxLDL” group (Fig. 5c). A similar result of Oil red O staining was observed when 1-MT was used. Therefore, before and after oxLDL treatment, using an IDO1 inhibitor to inhibit IFN-γ-induced IDO1 could decrease the degree of foaming.

In conclusion, before and after oxLDL treatment, IFN-γ-induced IDO1 exhibited different degrees of promotion on foaming, suggesting that the effect of IDO1 on foaming in the developmental process of atherosclerosis was greater than that in established atherosclerosis.

### Before and after oxLDL treatment, IFN-γ-induced IDO1 exhibited different degrees of promoting effects on cell apoptosis and inflammatory factor production

**Fig. 6:**
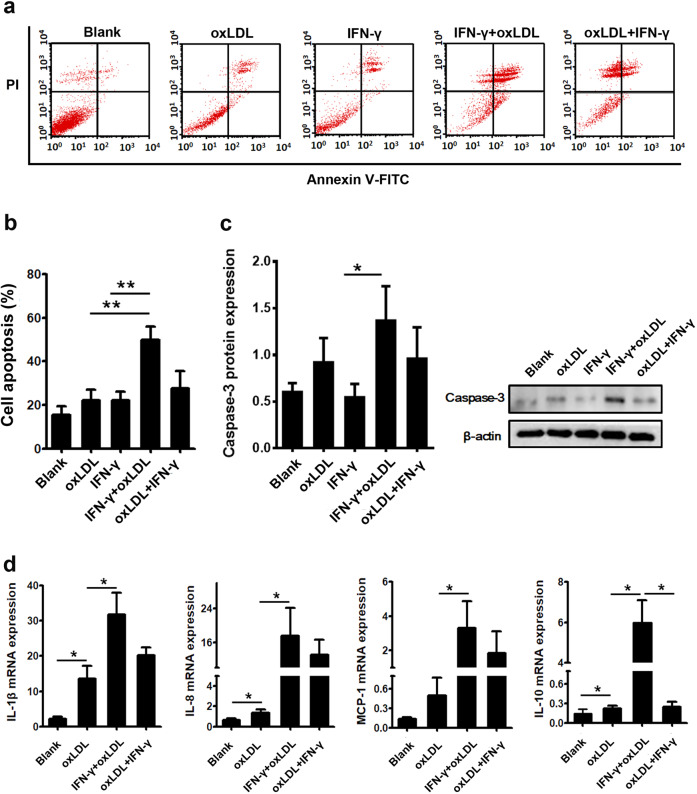
Before and after oxLDL treatment, IFN-γ-induced IDO1 exhibited different degrees of promotion on cell apoptosis and inflammatory factor production. **a** The degree of cell apoptosis was evaluated by flow cytometry. PI for nuclear staining and Annexin V-FITC for cytomembrane staining. The UL (upper left) quadrant represents cellular debris and damaged cells. The UR (upper right) and LR (lower right) quadrants represent apoptotic cells in the late or early stages. The LL (lower left) quadrant shows the normal cells. **b** Quantitative analysis of cell apoptosis. The total cell apoptosis is the sum of the percentage of late cell and early cell apoptosis. **c** Western blot analysis of caspase-3 expression in THP-M. **d** The mRNA levels of IL-10, MCP-1, IL-1β and IL-8 were measured by real-time PCR. Data were based on at least three independent experiments. *p < 0.05, **p < 0.01

The effect of IFN-γ-induced IDO1 on THP-M apoptosis before and after oxLDL treatment was investigated. The single treatment of “oxLDL” or “IFN-γ” slightly induced cell apoptosis. In addition, the cell apoptosis rate of the “IFN-γ+oxLDL” group was increased nearly two times compared with that of the “oxLDL” group. However, the cell apoptosis rate of the “oxLDL+IFN-γ” group was almost the same as that of the “oxLDL” group (Fig. 6a, b). To further confirm our results, the expression of caspase-3, a marker of cell apoptosis, was analyzed by western blots, and the results showed a similar trend as that of the cell apoptosis rate (Fig. 6c). In addition, we investigated the expression of cleaved caspase-3, another important marker for cell apoptosis, by western blot, which showed a similar result as that of caspase-3 (Supplementary Fig. S2). This result indicated that IFN-γ-induced IDO1 exhibited greater promoting effects on THP-M apoptosis before oxLDL treatment than after oxLDL treatment.

Furthermore, the effect of IFN-γ-induced IDO1 on inflammatory factor production before and after oxLDL treatment was investigated. Similar to the results shown in Fig. 3a, the levels of all inflammatory factors were increased in the “oxLDL” groups, which indicated that foam cell formation triggered an inflammatory response. The levels of all inflammatory factors in the “IFN-γ+oxLDL” group were significantly higher than those in the “oxLDL” group, indicating that IFN-γ-induced IDO1 exaggerated the inflammatory response (Fig. 6d). In addition, the production of inflammatory factors in the “oxLDL+IFN-γ” group was lower compared to the “IFN-γ+oxLDL” group (Fig. 6d). This result indicated that IFN-γ-induced IDO1 exhibited greater promotion on inflammatory factor production before oxLDL treatment in THP-M than after oxLDL treatment.

**Fig. 7:**
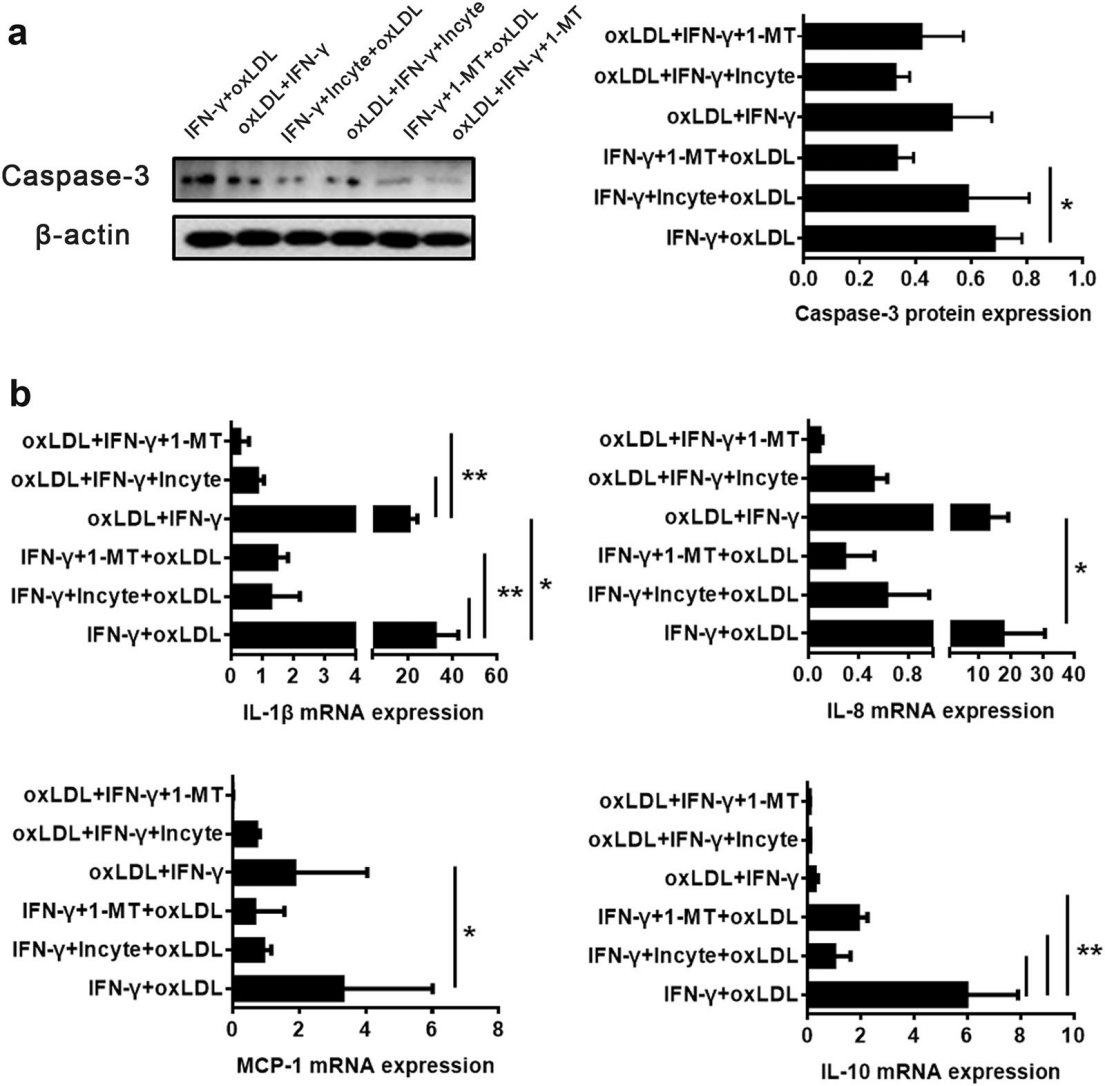
Before and after oxLDL treatment, the IFN-γ-mediated promotion of cell apoptosis and inflammatory factor production were reversed by IDO1 inhibition. **a** Western blot analysis of caspase-3 expression in THP-M. **b** The mRNA levels of IL-10, MCP-1, IL-1β and IL-8 were measured by real-time PCR. The designation of different treatments, such as IFN-γ+oxLDL, oxLDL+IFN-γ, IFN-γ+Incyte+oxLDL, oxLDL+IFN-γ+Incyte, IFN-γ+1-MT+oxLDL and oxLDL+IFN-γ+1-MT, was described in the Materials and Methods. Data were based on at least three independent experiments. *p < 0.05, **p < 0.01

Finally, the effects of the IDO1 inhibitor Incyte or 1-MT on IFN-γ-induced cell apoptosis and inflammatory factor production were tested. We observed that the combination treatments “IFN-γ+oxLDL” and “oxLDL+IFN-γ” increased the expression of caspase-3 (Fig. 6c). The enhanced caspase-3 expression was reversed by supplementation with Incyte or 1-MT, regardless of the order of treatment, before or after oxLDL treatment (Fig. 7a). Similarly, before and after oxLDL treatment, using an IDO1 inhibitor (Incyte or 1-MT) to inhibit IFN-γ-induced IDO1 also decreased the production of inflammatory factors (Fig. 7b).

In summary, IDO1 exhibited greater promoting effects on THP-M apoptosis and inflammatory factor production in the developmental process of atherosclerosis than in established atherosclerosis.

### The IDO1 inhibitor 1-MT could ameliorate the development of atherogenesis in high-fat diet (HFD)-fed ApoE^−/−^ mice

**Fig. 8:**
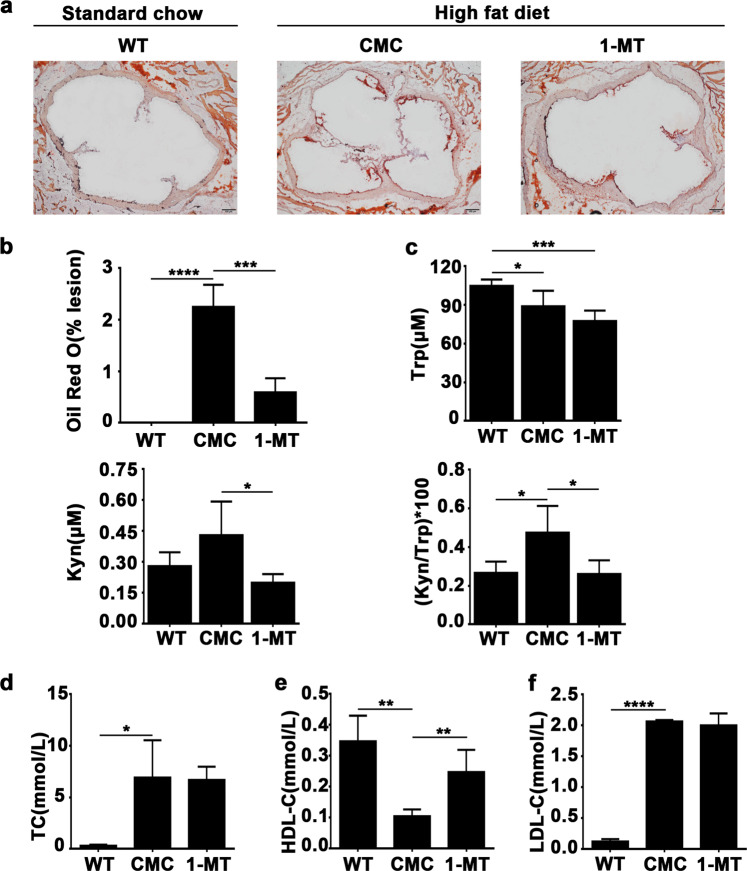
The IDO1 inhibitor 1-MT could ameliorate the development of atherogenesis in high-fat diet (HFD)-fed ApoE^−/−^ mice. Eight-week-old WT and ApoE^−/−^ mice were used and killed at 12 weeks of age. **a** Representative photomicrographs of aortic roots from 12-week-old mice of different groups stained with Oil red O. **b** The atherosclerotic plaque area/total aortic sinus area ratio. Atherosclerotic lesion areas were measured as the mean sizes of multiple plaques located in 5 sections in each mouse. **c** IDO1 activity analyzed by HPLC (WT group: *n* = 6, CMC group: *n* = 4, 1-MT group: *n* = 4). **d** Total cholesterol (TC). **e** High-density lipoprotein cholesterol (HDL-C). **f** Low-density lipoprotein cholesterol (LDL-C). *n* = 4. *p < 0.05, **p < 0.01, ***p < 0.001, ****p < 0.0001

For the in vivo assay, we investigated the potential effects of the IDO1 inhibitor 1-MT on preventing atherosclerotic lesion formation in ApoE^−/−^ mice. First, the effect of 1-MT on IDO1 inhibition in vivo was evaluated. As shown in Fig. 8c, the Trp concentration in the CMC group (ApoE^−/−^ mice administered 0.5% CMC and fed a HFD) was markedly lower than that in the WT group (C57BL/6 mice fed standard chow), and the Trp concentration in the 1-MT group (ApoE^−/−^ mice administered 1-MT and fed a HFD) was lower than that of the CMC group. The Kyn in the CMC group was higher than that in the WT group, and the administration of 1-MT significantly decreased the Kyn level compared with that in the CMC group. In the Kyn/Trp analysis, we found that the IDO1 activity in the CMC group was upregulated, while 1-MT could significantly reverse the enhanced IDO1 activity.

Next, the status of atherosclerotic lesion formation was explored by Oil red O staining.^50^ In the WT group, aortic atherosclerosis formation was seldom detected after 4 weeks of standard chow (Fig. 8a). Eight-week-old ApoE^−/−^ mice were fed a HFD for 4 weeks, and all mice formed atherosclerotic lesions (Fig. 8a). Treatment with 1-MT resulted in a significant decrease in the plaque area ratio (Fig. 8b). These results indicated that 8-week-old ApoE^−/−^ mice fed a HFD for 4 weeks could form atherosclerosis plaques and that 1-MT could attenuate atherosclerosis progression.

Hypercholesterolemia, defined by an overload of lipids, as well as an imbalance of lipoproteins, including low-density lipoprotein cholesterol (LDL-C) and high-density lipoprotein cholesterol (HDL-C), acts as a driving force and plays a vital role in the development and progression of atherosclerosis.^51,52^ Herein, the in vivo study showed that serum TC and LDL-C were significantly increased in ApoE^−/−^ mice fed a HFD^53^ (Fig. 8d, f), while serum HDL-C was decreased (Fig. 8e). Administration of 1-MT to ApoE^−/−^ mice significantly restored the level of HDL-C (Fig. 8e); however, it did not impact the levels of TC and LDL-C (Fig. 8d, f).

## Discussion

The exact role of IDO1, an immunosuppressive enzyme, in the developmental process of atherosclerosis has not been fully studied, nor has that of IDO1′s isozyme TDO. Here, using human aorta samples, the codistribution of IDO1/TDO and lymphocytes/macrophages was demonstrated in human atherosclerotic plaques for the first time, indicating the involvement of IDO1 and TDO in the immune responses in atherosclerosis.

Using blood samples of atherosclerotic patients, the expression and activity of IDO1 and TDO were found to vary with the grade of the histological classification of atherosclerosis patients. Notably, our study sample included patients with myocardial infarction. In contrast, the sampling of previous studies on the relationship between atherosclerosis and IDO1 usually ignored patients with myocardial infarction.^26,33,34^ The results showed that the expression and activity of IDO1 and TDO were increased in the early atherosclerosis stage (grade I, II), but the increase did not continue in advanced atherosclerosis (grade III). IDO1 and TDO were downregulated in the advanced atherosclerosis stage compared with that in the early atherosclerosis stage. Considering the documented atheroprotective function of IDO1 in established atherosclerosis, we hypothesized that high IDO1 and TDO expression and/or activity might be a self-protective mechanism for atherosclerosis in advanced stages and that the comparable downregulation of IDO1 and TDO in advanced atherosclerosis led to post-myocardial infarction.

Studies on the relationship between atherosclerosis and KP have mostly focused on the metabolites of tryptophan,^28,54^ and the association between Trp level and the developmental stages of atherosclerosis has rarely been investigated. Interestingly, in our study, a significant correlation between the concentration of Trp rather than Kyn and the pathological grading of atherosclerosis was observed. These results suggest that the depletion of Trp might be an important factor affecting the development of atherosclerosis. As an essential amino acid, Trp plays abundant and vital roles in many physiological and pathological processes, and its function in the pathologic progression of atherosclerosis deserves to be explored.

THP-M-derived foam cells are an in vitro model capable of studying atherogenesis. In the present study, we revealed for the first time that oxLDL could induce the expression of IDO1 via the PI3K/Akt/NF-κB pathway in THP-M, which indicated the potential function of IDO1 in foam cells. THP-M foam cell formation is a marker of established atherogenesis, so this result also indicated the function of IDO1 in established atherogenesis. To determine the role of IDO1 in the developmental process of atherosclerosis, the effects of several designated treatments, “oxLDL”, “IFN-γ”, “oxLDL+IFN-γ” and “IFN-γ+oxLDL”, on the expression of IDO1 and the degree of foaming were evaluated, as well as IDO1 inhibitor-mediated reversion of the effects of these treatments, were also investigated. We concluded that before and after atherosclerosis was established, IFN-γ-induced IDO1 exhibited different degrees of promoting effects on the degree of foaming. Similarly, before and after treatment with oxLDL, IFN-γ-induced IDO1 exhibited different degrees of promoting effects on THP-M cell apoptosis and inflammatory factor production. Therefore, IDO1 seemed to show greater promoting effects on the degree of foaming, cell apoptosis and inflammatory factor production in the developmental process of atherosclerosis than in established atherosclerosis. We concluded that IDO1 played a proatherosclerotic role in the developmental process of atherosclerosis.

A previous study has shown that inhibition of IDO1 can increase atherosclerosis in ApoE^−/−^ mice,^36^ which raises concerns for the potential pharmaceutical application of IDO1 inhibitors. In the present study, we revealed the proatherosclerotic function of IDO1 in the developmental stages of atherosclerosis. We found that IFN-γ-induced IDO1 could promote the transformation of THP-M to foam cells, and two classical IDO1 inhibitors, Incyte^55^ and 1-MT,^56^ could inhibit IFN-γ-induced IDO1 (ref. 57) and weaken the foaming. The in vivo study using ApoE^−/−^ mice showed that treatment with the IDO1 inhibitor 1-MT could significantly reverse the enhanced IDO1 activity and attenuate atherosclerosis progression in the early stage of atherosclerosis.

In conclusion, our data revealed the relationship between IDO1/TDO and atherosclerotic pathological grading and indicated that IDO1 and its isoenzyme TDO were important modulators of the immunoinflammatory responses in atherosclerosis. In atherosclerotic blood samples or foam cells, we confirmed that the expression and activity of IDO1 could accelerate the developmental process of atherosclerosis. The IDO1 inhibitor 1-MT could ameliorate the development of atherogenesis in high-fat diet (HFD)-fed ApoE^−/−^ mice. Therefore, inhibition of activated IDO1 could be a treatment for atherosclerosis, and IDO1 inhibitors have good prospects in the prevention of atherosclerosis.

## Materials and methods

### Human samples

Paraffin-embedded human aortic samples, including normal and atherosclerotic aortas, were provided by Dr. Saiyin Hexige from Fudan University. Fifty-one blood samples from patients with atherosclerosis and 10 blood samples from healthy volunteers were collected from Xinhua Hospital from February 2017 to December 2017. The baseline characteristics of the patients are listed in Table 2. The histological classification of the atherosclerosis patients was determined by Xinhua Hospital according to the degree of coronary artery stenosis: Grade I: 1–25% stenosis, Grade II: 26–50% stenosis, Grade III: 51–100% stenosis. Here, classification grades I and II were defined as early atherosclerosis, and grade III in post-myocardial infarction was defined as advanced atherosclerosis. All patients and healthy volunteers were well informed. The procedure of the experiment was approved by the Human Ethics Committee of Fudan University and Xinhua Hospital.

**Table 2:** Baseline characteristics of patients with atherosclerosis

Variables	Number
Gender	
Male	31
Female	20
Age	42–91
Pathological grades	
Grade (−)	10
Grade I	16
Grade II	18
Grade III	7

### H&E staining and immunostaining analysis

Paraffin sections were cut and stained with hematoxylin and eosin (H&E, Beyotime, China). The consecutive sections were used for immunostaining. After dewaxing with xylene and dehydrating with a gradient of concentration of alcohol, the slides underwent antigen retrieval in 10 mM citrate buffer (Beyotime, China) for 20 min at 100 °C. Nonspecific antigens and endogenous peroxidase were blocked with 3% H_2_O_2_ and serum. The slides were incubated with primary anti-IDO1 antibody (CST, USA, No. 86630), anti-TDO antibody (Proteintech, USA, No. 15880–1-AP), anti-CD3 antibody (Abcam, USA, No. ab699) and anti-CD68 antibody (Abcam, USA, No. ab955) diluted in PBS (1: 100) overnight at 4 °C. Slides were then incubated with secondary antibodies, Alexa Fluor 488 goat anti-rabbit (Invitrogen, USA, No. A-11034), Alexa Fluor 555 goat anti-rabbit (Invitrogen, USA, No. A32727), and DAPI (Sigma-Aldrich, USA, No. D9542) diluted in PBS (1:100) for 1 h at room temperature. The immunostaining analysis was carried out under a laser scanning confocal microscope (Carl Zeiss AG, LSM710, Germany).

### Human peripheral blood mononuclear cell isolation

A 7 mL Ficoll separation solution (1.077 g/mL, CAPRICORN, Germany) was added to a 15 mL EP tube. After anticoagulant, whole blood was added to PBS and diluted in equal proportion. Then, the solution was slowly dripped into the upper layer of separation liquid along the tube wall and centrifuged at 1200×*g* for 30 min. Next, the white cell layer was poured into the new EP tube and supplemented with PBS. Finally, human peripheral blood mononuclear cells were obtained after centrifugation at 300×*g* for 10 min.

### High-performance liquid chromatography (HPLC)

The total activity of IDO1 and TDO was evaluated by measuring the levels of Trp and Kyn^5^ by HPLC as previously reported. The culture medium from THP-1 cells was treated with 5% perchloric acid and methanol to remove protein, and the supernatants were subjected to HPLC analysis. The analysis was performed on an Agilent 1260 series HPLC system (Agilent Technologies, USA) equipped with a quaternary pump and a UV detector. HPLC analysis of the samples was performed using an Agilent C18 column (5 μm particle size, L × I.D. 25 cm × 4.6 mm) preceded by a C18 guard column (Dikma, China). The mobile phase was 15 mM acetic acid-sodium acetate buffer (pH 3.6) containing 6% acetonitrile by volume. The UV monitoring wavelengths of Trp and Kyn were 280 nm and 360 nm, respectively.

### RNA extraction and quantitative real-time PCR

Total RNA was extracted from the human peripheral blood mononuclear cells and THP-M with TRIzol reagent (TaKaRa, Japan), and the concentration of the obtained RNA was assessed using a Nano Drop 2000/2000c (Thermo Scientific, USA). RT-PCR was performed to synthesize cDNA using a Premium One-Step RT-PCR kit (TaKaRa, Japan). Real-time PCR was performed using a SYBR Green Mastermix kit (TaKaRa, Japan), and the results were analyzed using the Bio-Rad CFX Manager software, version 3.1.^58^ The primers used are listed in Table 3.

**Table 3:** Primers used for real-time PCR

Genes	Forward (5′–3′)	Reverse (5′–3′)
IL-1β	GCTGAGGAAGATGCTGGTTC	TCCATATCCTGTCCCTGGAG
IL-8	TAGCAAAATTFAFFCCAAGG	AAACCAAGGCACAGTGGAAC
MCP-1	ATCAATGCCCCAGTCACCT	AGTC TTCGGAGTTTGGGTTTG
IL-10	CCCTGTGAAAACAAGAGCAAGG	ACCCTGATGTCTCAGTTTCGT
IDO1	ATGCAAGAACGGGACACT	GCCTTTCCAGCCAGACAA
TDO	GGTGTGAATAGAGCCAGCAAAGGAG	AAGTCCAAGGCTGTCATCGTCTCCA
IDO2	TGAAGGAGTTTCCCAAGA	CATAACTGCGGTTCCACC
β-actin	CGGGAAATCGTGCGTGAC	GGAAGGAAGGCTGGAAGAG

### Western blot analysis

Total proteins were extracted by RIPA lysis buffer containing PMSF (Beyotime, China) and quantified with a standard BCA assay (Beyotime, China). Protein samples were separated by SDS-PAGE and then transferred to a PVDF membrane. Primary antibodies against IDO1 (CST, USA, No. 86630), TDO (Proteintech, USA, No. 15880–1-AP), pAkt/Akt (Abways, China, No. CY6569 and AB3147), pp65 (CST, USA, No. 3303), IKKα/β (Abways, China, No. CY3048), IκB (Abways, China, No. AB3178) and β-actin (HuaBio, China, No. ET1701–80) and Cleaved Caspase 3 (CST, USA, 9664T) were used and diluted 1000 times with Primary Antibody Dilution Buffer (Beyotime, China). Secondary antibodies were diluted 2000 times with blocking solution (5% nonfat dry milk dissolved in 1× PBS). The membranes were incubated with primary antibodies overnight at 4 °C and secondary antibodies for 1 h at 37 °C. Immunoreactive bands were visualized using the ECL kit (Tanon, China), and the integrated density of the bands was quantified by ImageJ software (NIH, USA).

### Cell culture and foam cell formation

THP-1 monocytes were grown at 37 °C in a 5% CO_2_ atmosphere to a density of 10^6^ cells/mL. The growth medium for THP-1 monocytes was RPMI 1640 (Gibco, USA) supplemented with 20% fetal bovine serum (FBS) (Gibco, USA), 50 units/mL penicillin, and 50 units/mL streptomycin. THP-1 monocytes (10^6^ cells/mL) in 6-well plates were treated with 100 nM PMA (Sigma, USA) for 3 days to differentiate into macrophages. Then, THP-1 macrophages (THP-M) were washed and further incubated in RPMI 1640 medium with 3% FBS and 25 mg/L oxLDL for 24 h to form foam cells.^59^

### Experimental conditions

THP-M were exposed to the following conditions: (a) blank (no treatment); (b) oxLDL (THP-M were treated with 25 mg/L oxLDL (Jingke Chemistry, China) for 24 h); (c) IFN-γ (THP-M were treated with 100 ng/mL IFN-γ (Sigma, USA) for 24 h); (d) IFN-γ+oxLDL (THP-M were pretreated with 100 ng/mL IFN-γ for 24 h, then treated with 25 mg/L oxLDL for 24 h); (e) oxLDL+IFN-γ (THP-M were pretreated with 25 mg/L oxLDL for 24 h, then treated with 100 ng/mL IFN-γ for 24 h); (f) IFN-γ+1-MT+oxLDL (THP-M were pretreated with 100 ng/mL IFN-γ for 24 h, then treated with 10 μM 1-MT for 24 h and followed by the treatment of 25 mg/L oxLDL for 24 h); (g) oxLDL+IFN-γ+1-MT (THP-M were pretreated with 25 mg/L oxLDL for 24 h, then treated with 100 ng/mL IFN-γ for 24 h and followed by treatment with 10 μM 1-MT for 24 h). (h) IFN-γ+Incyte (INCB024360)+oxLDL (THP-M were pretreated with 100 ng/mL IFN-γ for 24 h, then treated with 20 nM Incyte for 24 h and followed by treatment with 25 mg/L oxLDL for 24 h); (i) oxLDL+IFN-γ+Incyte (THP-M were pretreated with 25 mg/L oxLDL for 24 h, then treated with 100 ng/mL IFN-γ for 24 h and followed by the treatment of 20 nM Incyte for 24 h).

### Oil red O staining

Cells were fixed with 4% paraformaldehyde for 30 min. Cells were washed twice with PBS and then stained with filtered Oil Red O solution (60% Oil Red O dye and 40% water; Sigma, USA) at room temperature for 30 min. Cells were washed twice with PBS and then washed with 60% isopropyl alcohol once. Oil red O staining was observed under a microscope (Olympus, Japan), and the intensity was measured by Image-Pro Plus 6.0^58^.

### Flow cytometry analysis of apoptosis

Cell apoptosis was analyzed by an Annexin V-FITC/PI apoptosis detection kit (Yeasen Biotech, China). Cells were harvested and then washed twice with PBS. The cell pellet was suspended in 1× binding buffer and supplemented with Annexin V-FITC and PI. The cells were gently vortexed and incubated for 15 min at room temperature in the dark, then supplemented with 400 μL 1× binding buffer and submitted to flow cytometry analysis using a FACSCalibur flow cytometer equipped with Cell Quest software (BD Biosciences, USA).^59^

### Animals and diets

Eight-week-old male wild-type and ApoE^−/−^ mice on C57BL/6 were purchased from Shanghai Model Organisms Center, Inc. (Shanghai, China) and bred in specific pathogen-free conditions. Mice were divided into 3 groups: WT group, wild-type C57BL/6 mice were fed a standard chow, *n* = 10; 1-MT group: ApoE^−/−^ mice were intragastrically administered 1-MT (100 mg/kg, 0.5% CMC as solvent) once daily and fed a high-fat diet (HFD) containing 21% fat and 0.15% cholesterol (Beijing Keao Xieli Feed Co., Ltd, Beijing, China), *n* = 9; CMC group, ApoE^−/−^ mice were intragastrically administered an equivalent volume of 0.5% CMC once daily and fed an HFD, *n* = 8. The administration lasted 4 weeks. All animal experimental procedures were approved by the Experimental Animal Committee of China of the Fudan University.

### Tissue and organ harvesting

At 12 weeks of age, mice were killed, and blood samples were collected and centrifuged to isolate the serum that was stored at −80 °C. The serum concentrations of Kyn and Trp were analyzed by HPLC, and the ratio of Kyn/Trp was calculated. Levels of Trp and Kyn in serum were analyzed by high performance liquid chromatography. The connective tissues extending from the point at which the aorta emerged from the heart to the aortic arch were carefully disserted and then embedded in 4% paraformaldehyde. Twenty-four hours later, the tissues were embedded in optimal cutting temperature (OCT) compound for cryostat sectioning.

### Quantification of atherosclerotic lesions

Hearts were perfused with physiological saline via an injector inserted into the left ventricle (outflow via an incision in the right atria) and then frozen in OCT. Eight-micrometer cryosections were taken of the aortic root, and multiple plaques located in 5 sections in each mouse were stained with Oil red O (Nanjing Jiancheng Bioengineering Institute, Nanjing, China). The lesion area fraction was calculated by dividing the lesion area by the area of the aortic wall and expressed as a percentage.

### Detection of TC, HDL-C, and LDL-C levels

TC, HDL-C, and LDL-C levels in serum were determined using an assay kit (Nanjing Jiancheng Bioengineering Institute, Nanjing, China) according to the manufacturers’ instructions.

### Statistical analysis

All the results are presented as the mean ± standard deviation (SD) and analyzed by one-way analysis of variance (ANOVA), followed by post hoc analysis using GraphPad Prism 5.0 (GraphPad, USA). A *p-value < 0.05 was considered statistically significant.

## Supplementary information

Online Data Supplement
