# Supplementary material for: The proatherosclerotic function of indoleamine 2, 3-dioxygenase 1 in the developmental stage of atherosclerosis
Source: Signal Transduct Target Ther. 2019 Jul 19;4:23. doi: 10.1038/s41392-019-0058-5 (PMC6799842; doi:10.1038/s41392-019-0058-5)
Supplement: Supplementary file 1 — Online Data Supplement [file 41392_2019_58_MOESM1_ESM.docx]

**Online Data Supplement for**

**The pro-atherosclerotic function of indoleamine 2, 3-dioxygenase 1 in the developmental stage of atherosclerosis**

Heng Liang^1#^, Mantian Chen^2#^, Fangfei Qi^1#^, Lei Shi^1^, Zhenzhen Duan^1^, Ruoyu Yang^1^, Jinchao He^1^, Bin Lou^3^, Yigang Li^2^*, Qing Yang^1^*

^1^ State Key Laboratory of Genetic Engineering, Department of Biochemistry, School of Life Sciences, Fudan University, Shanghai, China

^2^ Department of Cardiovascular Diseases, Xinhua Hospital, School of Medicine, Shanghai Jiaotong University, Shanghai, China

^3^ School of Pharmacy, Fudan University, Shanghai, China

*Correspondence: Qing Yang ([yangqing68@fudan.edu.cn](mailto:yangqing68@fudan.edu.cn)) and Yigang Li ([liyigang@xinhuamed.com.cn](mailto:dev@null))

^#^These authors contributed equally: Heng Liang, Mantian Chen and Fangfei Qi

**Figure S1 (related to Figure 4)**


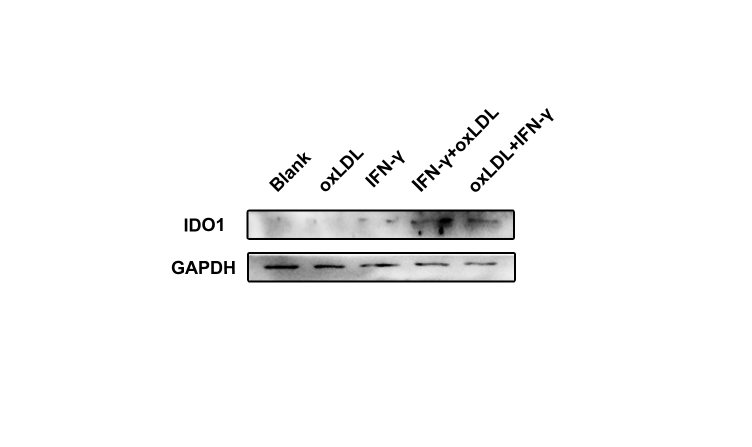


**Figure S1.** Induction of IDO1 in THP-M by different treatment was detected by western blot. The designation of different treatments such as oxLDL, IFN-γ, IFN-γ+oxLDL, oxLDL+IFN-γ, was described in the Materials and Methods.

**Figure S2 (related to Figure 6)**


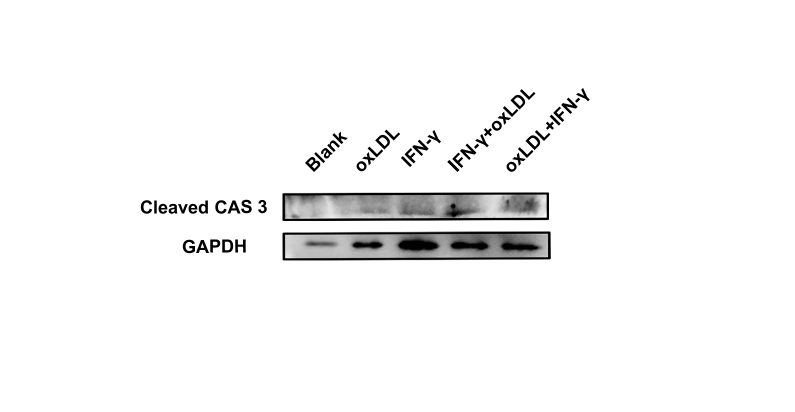


**Figure S2.** Western blot analysis of the expression of cleaved caspase-3 (cleaved CAS 3) in THP-M. The designation of different treatments such as oxLDL, IFN-γ, IFN-γ+oxLDL, oxLDL+IFN-γ, was described in the Materials and Methods.
